# Observation of Thickness-Modulated Out-of-Plane Spin–Orbit Torque in Polycrystalline Few-Layer Td-WTe_2_ Film

**DOI:** 10.3390/nano15100762

**Published:** 2025-05-19

**Authors:** Mingkun Zheng, Wancheng Zhang, You Lv, Yong Liu, Rui Xiong, Zhenhua Zhang, Zhihong Lu

**Affiliations:** 1 State Key Laboratory of Advanced Refractories, Wuhan University of Science and Technology, Wuhan 430081, China; zhengmingkun1993@wust.edu.cn (M.Z.); zhangwancheng@wust.edu.cn (W.Z.); 2 School of Materials, Wuhan University of Science and Technology, Wuhan 430081, China; 3 State Key Laboratory of Rare Earth Resource Utilization, Changchun Institute of Applied Chemistry, Chinese Academy of Sciences, Changchun 130022, China; lvyou@ciac.ac.cn; 4 Key Laboratory of Artificial Micro- and Nano-Structures of Ministry of Education, School of Physics and Technology, Wuhan University, Wuhan 430072, China; liuyong@whu.edu.cn (Y.L.); xiongrui@whu.edu.cn (R.X.)

**Keywords:** Td-WTe_2_, spin–orbit torque, spin Hall conductivities, magnetoresistance, spin diffusion length

## Abstract

The low-symmetry Weyl semimetallic Td-phase WTe_2_ exhibits both a distinct out-of-plane damping torque ($\tau_{\mathrm{DL}}$) and exceptional charge–spin interconversion efficiency enabled by strong spin-orbit coupling, positioning it as a prime candidate for spin–orbit torque (SOT) applications in two-dimensional transition metal dichalcogenides. Herein, we report on thickness-dependent unconventional out-of-plane $\tau_{\mathrm{DL}}$ in chemically vapor-deposited (CVD) polycrystalline Td-WTe_2_ (*t*)/Ni_80_Fe_20_/MgO/Ti (Td-WTN-*t*) heterostructures. Angle-resolved spin-torque ferromagnetic resonance measurements on the Td-WTN-12 structure showed significant spin Hall conductivities of *σ*_SH,y_ = 4.93 × 10^3^ (ℏ/2e) Ω^−1^m^−1^ and *σ*_SH,z_ = 0.81 × 10^3^ (ℏ/2e) Ω^−1^m^−1^, highlighting its potential for wafer-scale spin–orbit torque device applications. Additionally, a detailed examination of magnetotransport properties in polycrystalline few-layer Td-WTe_2_ films as a function of thickness revealed a marked amplification of the out-of-plane magnetoresistance, which can be ascribed to the anisotropic nature of charge carrier scattering mechanisms within the material. Spin pumping measurements in Td-WTN-*t* heterostructures further revealed thickness-dependent spin transport properties of Td-WTe_2_, with damping analysis yielding an out-of-plane spin diffusion length of *λ*_SD_ ≈ 14 nm.

## 1. Introduction

A fundamental requirement in developing next-generation magnetic random-access memory (MRAM) is the concurrent achievement of high-speed magnetization switching and ultra-low power consumption [1,2]. Generally, conventional spin-transfer torque (STT)-MRAM employs spin-transfer torque from polarized currents for memory operations, but suffers from low spin polarization efficiency and inseparable read/write paths that increase power dissipation [3,4]. Remarkably, the development of spin–orbit torque (SOT)-MRAM has demonstrated remarkable advantages, including fully isolated read/write paths and superior power efficiency compared to conventional STT-MRAM, establishing it as a promising candidate for future memory applications [5,6]. The SOT-MRAM utilizes spin-orbit coupling in nonmagnetic layers to convert charge current to spin current through the spin Hall effect (SHE) or the Rashba–Edelstein effect (REE) [7,8]. The resulting spin accumulation at the ferromagnetic layer (FL) interface generates SOT for reliable magnetization reversal. However, SOT devices employing conventional heavy metal (Pt, Ta, W, etc.)/FL heterostructures are fundamentally restricted to in-plane spin polarization, requiring external magnetic fields for magnetization switching [9,10,11]. This intrinsic limitation highlights the necessity for designed strong spin–orbit coupling materials with pronounced out-of-plane spin-polarized spin current to realize field-free magnetization reversal in next-generation spintronics.

Two-dimensional transition metal dichalcogenides (TMDs) demonstrate remarkable potential for SOT devices [12], featuring strong SOC [13], non-trivial band topology [14], and long spin diffusion lengths [15], while their low crystal symmetry enables deterministic out-of-plane damping-like torque ($\tau_{\mathrm{DL}}$)-an uncommon synergy that surpasses conventional heavy metals [16]. For instance, Husain et al. reported a superior charge-to-spin conversion efficiency (0.25) in TaS_2_ (0.88 nm)/Permalloy (Py) (7 nm) heterostructures, outperforming other TMD-based SOT devices and setting a new benchmark for SOT performance in this material class [17]. Stiehl et al. first identified out-of-plane antidamping torque in *β*-MoTe_2_-based heterostructures, challenging the conventional understanding of SOT systems [18]. Xu et al. demonstrated efficient perpendicular magnetization switching in PtTe_2_/Au/CoTb heterostructures, achieving spin Hall conductivities comparable to those of platinum (0.2–2 × 10^5^ ℏ/2e Ω^−1^m^−1^) [19]. Guimaraes et al. employed spin-torque ferromagnetic resonance (ST-FMR) measurements on NbSe_2_/Py bilayers, revealing both out-of-plane field-like torque ($\tau_{\mathrm{FL}}$) and in-plane $\tau_{\mathrm{DL}}$ [20]. Also, Lv et al. explored the electric field modulation of SOT in WS_2_/NiFe bilayers and demonstrated that applying a back-gate voltage effectively controls the ratio between $\tau_{\mathrm{FL}}$ and $\tau_{\mathrm{DL}}$ [21].

Among them, the semimetallic Td-phase WTe_2_ emerges as a preeminent candidate among TMDs for efficient charge-to-spin conversion, owing to its unique combination of strong SOC and structural inversion symmetry breaking characteristics stemming from its type II Weyl semimetal nature that promotes robust spin–momentum locking and topological surface states [22]. Therefore, these distinctive properties of Td-WTe_2_ enable field-free magnetization switching in perpendicularly magnetized systems. MacNeill et al. identified an out-of-plane $\tau_{\mathrm{DL}}$ in WTe_2_/Py bilayers under current applied along the low-symmetry axis, but absent along the high-symmetry axis, highlighting crystal symmetry as a means to control SOT [23]. Additionally, their analysis of current-induced torques revealed that the $\tau_{\mathrm{DL}}$ scales exclusively with WTe_2_ thickness, while $\tau_{\mathrm{FL}}$ also exhibits significant thickness dependence [24]. Peng et al. demonstrated that magnetron-sputtered amorphous WTe_2_-based heterostructures achieve a remarkably high damping-like SOT efficiency of approximately 0.20, rivaling crystalline WTe_2_ systems while offering superior fabrication scalability [25]. Li et al. reported a significant enhancement of spin conductivity in WTe_2_, where the spin-hole locking effect at low temperatures substantially amplifies the field-like torque in WTe_2_/Py bilayers [22]. Wang et al. studied the PtTe_2_/WTe_2_ bilayer system and achieved the first room-temperature observation of a strong out-of-plane $\tau_{\mathrm{DL}}$, arising from the intrinsic crystalline asymmetry of WTe_2_, which facilitated perpendicular magnetization switching without the need for an external magnetic field [26]. According to the aforementioned research, SOT devices based on Weyl semimetal WTe_2_ can generate unconventional out-of-plane $\tau_{\mathrm{DL}}$, enabling perpendicular magnetization switching, making them a novel and competitive candidate in the field of spintronics. Currently, SOT devices based on Td-WTe_2_ are primarily fabricated using mechanical exfoliation, which preserves crystallographic orientation integrity but fails to meet the scalability requirements for spintronic device production [23,24,27,28]. However, high-quality centimeter-scale Td-WTe_2_ films are typically synthesized via chemical vapor deposition (CVD) [29,30,31], establishing CVD-grown polycrystalline few-layer Td-WTe_2_ as an ideal platform for wafer-scale spin–orbit torque (SOT) device exploration.

In this study, high-quality, centimeter-scale polycrystalline few-layer Td-WTe_2_ thin films with tunable c-axis orientation were successfully fabricated through a two-step process involving initial magnetron sputtering, followed by tellurium-assisted CVD. We systematically characterized the anisotropic magnetoresistance (MR) in polycrystalline few-layer Td-WTe_2_ films across 10–300 K, observing an enhanced out-of-plane MR compared to in-plane MR at 10 K, which is consistent with anisotropic scattering associated with spin–orbit coupling. Spin pumping measurements in Td-WTe_2_ (4, 6, 8, 10, and 12 nm)/Ni_80_Fe_20_ (NiFe)/MgO/Ti heterostructures reveal thickness-dependent damping enhancement, yielding a spin diffusion length of *λ*_SD_ ≈ 14 nm via inverse spin Hall effect (ISHE) analysis. More importantly, the angle-resolved ST-FMR measurements demonstrate thickness-dependent in-plane and out-of-plane $\tau_{\mathrm{DL}}$ spin Hall conductivities and spin Hall angle in Td-WTe_2_/NiFe devices, enabling precise control of the unconventional out-of-plane $\tau_{\mathrm{DL}}$ through thickness modulation.

## 2. Experimental Methods

### 2.1. Preparation Methods

We first fabricated WO_x_ (*x* < 3) films (3, 5, 7, 9, and 11 nm) on SiO_2_/Si substrates (HF-Kejing, Hefei, China) by RF magnetron sputtering at room temperature using a WO_3_ (99.99%, MAT-CN, Nanchang, China) target. The process maintained 1.2 Pa working pressure through controlled Ar flow, with 40 W sputtering power. Subsequently, a ceramic crucible loaded with 0.4 g of high-purity Te powder (99.999%, Aladdin, Wuhan, China) and a suitable quantity of molecular sieve (CanNa_12-2n_[(AlO_2_)_12_(SiO_2_)_12_]-*x*H_2_O (Aladdin, Wuhan, China)), which effectively regulates the release of Te vapor, was positioned at the center of the first heating zone in the CVD tube furnace. The temperature was then raised to 540 °C within 15 min under precisely controlled conditions. Simultaneously, the sputtered WO_x_ (*x* < 3) thin films were positioned in the second heating zone of the CVD tube furnace, located 24 cm downstream from the Te powder, and the temperature was elevated to 600 °C within 15 min under precisely controlled conditions. The WTe_2_ films were synthesized using CVD at atmospheric pressure for 60 min using high-purity H_2_ (40 sccm) and Ar (30 sccm) as carrier gases, with the specific CVD setup illustrated in Figure S1a. Prior to thermal deposition, the system was purged for 15 min with Ar (500 sccm) and H_2_ (200 sccm) to maintain oxygen-free growth conditions.

### 2.2. Device Fabrication

Firstly, NiFe with a thickness of 6 nm was deposited onto Td-WTe_2_ films of varying thicknesses (4, 6, 8, 10, and 12 nm) via magnetron sputtering technology. Subsequently, a MgO/Ti bilayer structure (2 nm each) (MgO(2)/Ti(2)) was deposited sequentially using magnetron sputtering. For the fabrication of spin pumping devices, Td-WTe_2_ samples were deposited onto SiO_2_ (300 nm)/Si with dimensions of 2 × 7 mm. The NiFe(6)/MgO(2)/Ti(2) layers were sputter-deposited onto the central 2 × 5 mm area of the WTe_2_ sample, leaving a reserved area for electrode connection. For the ST-FMR devices, multiple microstrip devices measuring 20 × 4 μm at various angles were fabricated on 1 × 1 cm Td-WTe_2_(*t*)/NiFe(6)/MgO(2)/Ti(2) (Td-WTN-*t*) samples using a positive photoresist (AZ5214) photolithography process in conjunction with Ar-ion milling technology. Subsequently, the top electrode pattern was defined using a negative photoresist lithography process, followed by the deposition of Ti (5 nm)/Au (50 nm) layers onto the patterned regions via magnetron sputtering to fabricate the top electrodes for device measurement connections.

### 2.3. Characterization Methods and Instruments

The structural properties and thickness of the film were evaluated using a SmartLab 3 kW X-ray diffraction (XRD) instrument with Cu-Kα radiation and X-ray reflectivity (XRR). The crystallographic phase of the material was characterized with a Renishaw Invia Qontor confocal Raman spectrometer (532 nm laser). The surface morphologies of the samples were analyzed using a SINICO-XK-40 optical microscope (MO) and a Bruker atomic force microscope (AFM). The compositional analysis of the samples was conducted using X-ray photoelectron spectroscopy (XPS) with a monochromatic Al-*K_α_* (1486.6 eV) X-ray source, employing an ESCALAB 250Xi system. The electrical resistance and magnetotransport properties of the material were measured using the Quantum Design Physical Property Measurement System (PPMS). The spin pumping system primarily consists of a PPMS for generating a magnetic field (−0.4 T to 0.4 T), an RF microwave source, an SR830 lock-in amplifier, and a 2182 nanovoltmeter. The RF alternating excitation is modulated by a sinusoidal signal (2 V) generated by the lock-in amplifier (171.4 Hz), with the magnetic field applied in the out-of-plane direction. The sample was placed at the center of a controllable angle rotation stage, and angle-dependent ST-FMR measurements were performed within a magnetic field range of −0.1 T to 0.1 T. By maintaining a fixed RF current (*I*_rf_) (5–9 GHz), the in-plane magnetic field was systematically scanned at a specific angle. The RF current modulation was produced by a microwave signal generator operating at an output power of 25 dBm, and the resulting voltage signal was measured using a lock-in amplifier with a frequency of 431.12 Hz.

## 3. Results and Discussions

**Figure 1:**
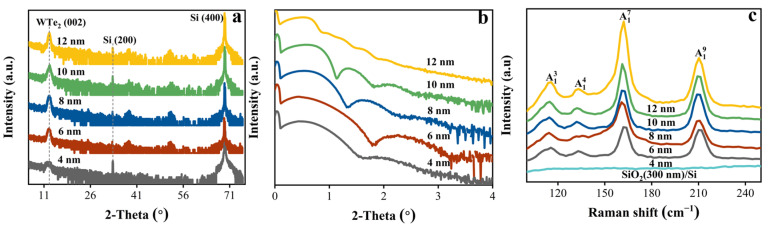
(**a**,**b**) XRD diffraction patterns and XRR spectra (intensity vs. 2*θ*) of polycrystalline few-layer Td-WTe_2_ films with varying thicknesses. (**c**) Raman spectra of polycrystalline few-layer Td-WTe_2_ films with different thicknesses, along with the SiO_2_/Si substrate.

The characteristic diffraction peak of the (002) crystal plane at 2*θ* = 12.61° for Td-WTe_2_ films with different thicknesses indicates that the polycrystalline few-layer Td-WTe_2_ grows along the c-axis direction of the crystal, as shown in Figure 1a. The thicknesses of Td-WTe_2_ films were accurately determined to be 3.96 nm, 6.12 nm, 8.05 nm, 9.94 nm, and 12.02 nm by fitting the XRR spectra using X’Pert Reflectivity software, showing excellent agreement with the nominal values (Figure 1b). The Raman spectrum in Figure 1c reveals the characteristic resonance modes $A_{1}^{3}$, $A_{1}^{4}$, $A_{1}^{7}$, and $A_{1}^{9}$ at 114.8, 132.6, 162.1, and 210.1 cm^−1^, respectively, consistent with previous reports [32]. The dominant $A_{1}^{7}$ peak at 162.1 cm^−1^ unequivocally confirms that all synthesized WTe_2_ thin-film samples exhibit the Td-phase structure. Furthermore, Figure S1b,c displays the MO and AFM images of the polycrystalline few-layer Td-WTe_2_ film (4 nm) grown by CVD, illustrating its uniform coverage on the Si/SiO_2_ (1 × 1 cm^2^) substrate with a surface roughness of ~0.92 nm. The XPS spectrum of the 4 nm thick Td-WTe_2_ film exhibits characteristic peaks at 31.41 eV (W 4f_7/2_), 33.53 eV (W 4f_5/2_), 572.81 eV (Te 3d_5/2_), and 583.17 eV (Te 3d_3/2_), confirming the presence of W^4+^ and Te^2−^, as shown in Figure S1d,e.

**Figure 2:**
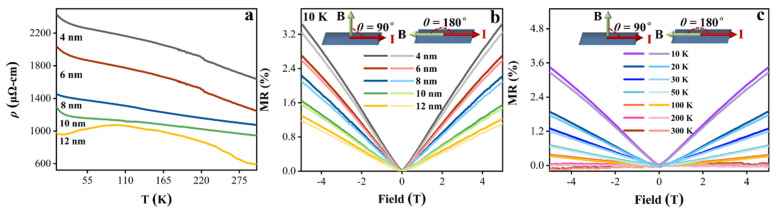
(**a**) The *ρ* variation of polycrystalline few-layer Td-WTe_2_ (4, 6, 8, 10, and 12 nm) from 10 to 300 K. (**b**) The MR of polycrystalline few-layer Td-WTe_2_ (4, 6, 8, 10, and 12 nm) under out-of-plane and in-plane magnetic field configurations at 10 K. (**c**) The MR of Td-WTe_2_ (4 nm) under in-plane and out-of-plane magnetic fields from 10 to 300 K.

Figure 2a shows the resistivity (*ρ*) of polycrystalline few-layer Td-WTe_2_ measured from 10 K to 300 K using the four-point probe technique, where *ρ* exhibits a linear relationship with temperature [33]:

(1) $$\rho =\rho_{0}+aT+bexp(-\Delta /T)$$

Here, *ρ*_0_ represents the residual resistivity, *a* is the coefficient for electron–phonon scattering, *b* corresponds to the nonlinear contribution from electron–electron interactions, and Δ denotes the activation temperature or energy gap associated with nonlinear excitations. The *ρ* of Td-WTe_2_ films increases with decreasing thickness, which is closely related to the enhanced effects of defects, impurities, and interface scattering in thinner films, as shown in Figure 2a. Moreover, all Td-WTe_2_ samples exhibit a metal–insulator transition, with *ρ* increasing at lower temperatures due to localization effects, consistent with their semimetallic character [34,35]. Additionally, Table S1 summarizes the *ρ* and the conductivity (*σ*) measurements of Td-WTe_2_ films with thicknesses of 4, 6, 8, 10, and 12 nm.

Figure 2b,c shows the magnetotransport properties of polycrystalline few-layer Td-WTe_2_ investigated by measuring the MR variations under in-plane and out-of-plane magnetic fields (B = 5 T). The MR values for Td-WTe_2_ samples with different thicknesses were calculated using Equation (2).

(2) $$\mathrm{MR}=\frac{R(B)-R(0)}{R(0)}\times 100\%$$

Here, *R*(*B*) denotes the resistance under an applied magnetic field, whereas *R*(0) represents the resistance at a zero magnetic field. As shown in Figure 2b,c, all samples exhibit positive, non-saturating MR at 10 K, which is significantly smaller than that of single-crystal Td-WTe_2_, likely attributed to the increased defects and disorder in polycrystalline films [36]. Compared to thicker samples, the 4 nm thick Td-WTe_2_ demonstrates a higher MR value, which is likely due to the reduced carrier mobility in the thin layers of Td-WTe_2_ and the imbalance in charge compensation between electron and hole densities [37]. Furthermore, we observed that the MR is higher under an out-of-plane magnetic field compared to an in-plane magnetic field, which can be ascribed to the anisotropic scattering in Td-WTe_2_ [38,39]. Figure 2c demonstrates that the MR of Td-WTe_2_ (4 nm) increases as the temperature decreases, reaching a value of 3.46% at 10 K.

**Figure 3:**
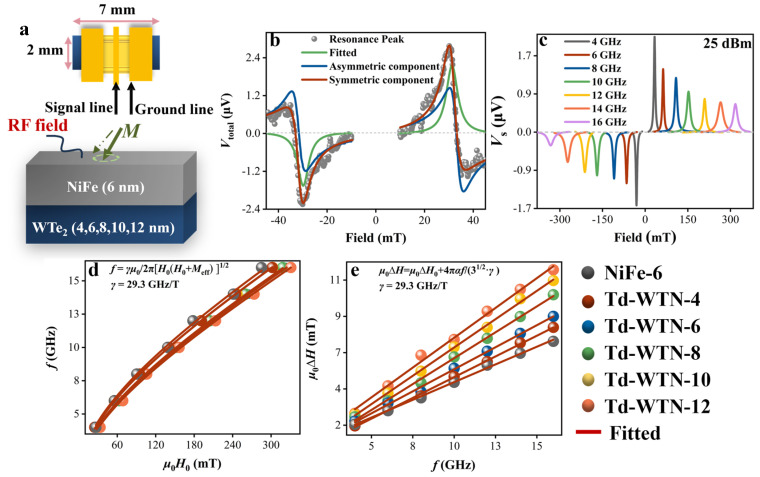
(**a**) Schematic representation of the spin-pumping experimental mechanism. (**b**) *V*_total_ spectrum of the Td-WTN-4 sample at 4 GHz, with *V*_s_ and *V*_a_ as fitted curves. (**c**) The *V*_s_ of Td-WTN-4 at different frequencies. (**d**,**e**) The *H*_0_ curves and Δ*H* versus *f* curves for Td-WTN-*t* and NiFe(6). The solid spheres represent the experimentally measured data, while the red line indicates the fitted curve.

Figure 3a depicts the spin pumping measurement process conducted on the Td-WTN-*t* samples to characterize their spin transport properties, with the detailed experimental procedure available in our previous report [40]. We measured the mixed voltage (*V*_total_) generated by the conversion of spin current, injected from the NiFe layer, into charge current within the Td-WTe_2_ layer via the ISHE, resulting from the spin pumping effect. Figure 3b illustrates the *V*_total_ of the Td-WTN-4 sample measured at 4 GHz and 25 dBm. By applying Equation (3), the symmetric Lorentzian profile (green line), associated with the bulk ISHE and Seebeck effects, and the asymmetric Lorentzian line (blue line), resulting from the anisotropic magnetoresistance and anomalous Hall effect in the NiFe layer, were fitted [41]:

(3) $$V_{\mathrm{total}}=\frac{V_{s}\Delta H^{2}}{\Delta H^{2}+{\left(H_{\mathrm{ext}}-H_{0}\right)}^{2}}+\frac{V_{a}(H_{\mathrm{ext}}-H_{0})\Delta H}{\Delta H^{2}+{\left(H_{\mathrm{ext}}-H_{0}\right)}^{2}}$$

(4) $$V_{\mathrm{SE}}=(V_{s}(+H_{0})-V_{s}(-H_{0}))/2$$

Among them, *V*_s_ represents the symmetric Lorentz voltage, *V*_a_ represents the antisymmetric Lorentz voltage, Δ*H* denotes the linewidth, *H*_0_ is the resonance field, and *H*_ext_ is the externally applied magnetic field. To extract the pure spin–charge conversion voltage (*V*_SE_), *V*_S_ is corrected according to Equation (4) to eliminate the influence of the Seebeck effect [42]. Figure 3c displays *V*_S_ of 4 nm thick Td-WTe_2_ under positive and negative magnetic fields in the frequency range of 4–16 GHz, with *V*_SE_ values extracted from *V*_s_ using Equation (4), while also revealing that the *V*_s_ value of Td-WTN-4 decreases as the frequency (*f*) increases.

To determine the effective saturation magnetization (*M*_eff_) of Td-WTN-*t*, the Kittel formula was employed to fit the relationship between *H*_0_ and *f* in Figure 3d, as expressed by Equation (5) [43]:

(5) $$f=\frac{\gamma \mu_{0}}{2\pi }\sqrt{H_{0}(H_{0}+M_{\mathrm{eff}})}$$

Here, $\gamma =\frac{g\mu_{B}}{\hbar }$ represents the gyromagnetic ratio, *g* is the Landé splitting factor, which is 2.1, $\mu_{B}$ is the Bohr magneton, ℏ is the reduced Planck constant, and $\mu_{0}$ is the vacuum permeability. Table 1 summarizes the *M*_eff_ values of the Td-WTe_2_-*t* and NiFe(6) samples. The results reveal that *M*_eff_ decreases progressively with increasing Td-WTe_2_ thickness, reaching a maximum of 549 kA/m, which is close to the saturation magnetization (*M*_s_). This trend is attributed to the modulation of interfacial magnetism in the NiFe(6) layer induced by the Td-WTe_2_ overlayer [25]. Table 1 also shows a slight decrease in *M*_s_ with increasing WTe_2_ thickness, which may be attributed to the influence of WTe_2_ on the WTe_2_/NiFe interface, leading to modifications in the electronic structure of the NiFe layer. Meanwhile, Figure S2a displays the VSM data for each layer of the Td-WTN-4 sample, demonstrating that Td-WTe_2_ is uniformly deposited on the NiFe layer. Furthermore, XRR analysis of the Td-WTe_2_(6)/NiFe(6) bilayer in Td-WTN-6 revealed a uniform 6.08 nm NiFe film atop a 5.86 nm Td-WTe_2_ layer, further confirming precise thickness control and a layer-by-layer growth mode (Figure S2b). Figure 3e demonstrates the linear dependence of Δ*H* on *f* for Td-WTN-*t*, indicating that Gilbert damping is the primary contributing factor [44]. The Gilbert damping (*α*) values for Td-WTN-*t* with varying thicknesses are extracted based on the Landau-Lifshitz-Gilbert (LLG) equation, as shown in Equation (6) [45].

(6) $$\mu_{0}\Delta H=\mu_{0}\Delta H_{0}+\frac{4\pi }{\sqrt{3}\gamma }\alpha f$$

Here, Δ*H*_0_ is the inhomogeneous line-broadening factor. Table 1 demonstrates that as the thickness of WTe_2_ ($t_{{\mathrm{WTe}}_{2}}$) in Td-WTN-*t* increases, *α* gradually rises, and *H*_0_ shifts towards higher magnetic fields (Figure S3), likely due to the enhanced SOC effects in thicker Td-WTe_2_. It is important to emphasize that the Δ*H*_0_ values obtained from fitting all samples using Equation (6) are minimal, demonstrating that the CVD-grown Td-WTe_2_/NiFe heterostructure exhibits exceptional quality. Furthermore, determining the effective mixing conductance ($g_{\mathrm{eff}}^{↑↓}$) is also a critical parameter for evaluating the efficiency of spin pumping, as shown in Equation (7) [46]:

(7) $$g_{\mathrm{eff}}^{↑↓}=\frac{4\pi M_{s}t_{\mathrm{NiFe}}}{g\mu_{B}}(\alpha -\alpha_{0})$$

where *α*_0_ is the damping constant of NiFe (6), and Δ*α* = *α* − *α*_0_represents the change in damping relative to the NiFe layer, as shown in Table 1. Our measurements reveal that Td-WTN-*t* (*t* > 8) exhibits an exceptionally large $g_{\mathrm{eff}}^{↑↓}$, exceeding typical values for high-SOC TMDs by an order of magnitude (Table 1) [47,48,49], while reaching magnitudes comparable to heavy metals like Pt and Ta [50,51]. Notably, the $g_{\mathrm{eff}}^{↑↓}$ in Td-WTN-*t* increases with $t_{{\mathrm{WTe}}_{2}}$, likely due to enhanced spin pumping and suppressed spin backflow in thicker flakes [52].

Based on the ballistic transport theory, we determined the *α* mechanism of the Td-WTe_2_-*t* samples, enabling the derivation of the spin diffusion length (*λ*_SD_) along the thickness direction of polycrystalline Td-WTe_2_ prepared using CVD, as shown in Equation (8) [53].

(8) $$\alpha =\alpha_{0}+\frac{g\mu_{B}g_{r}^{↑↓}}{4\pi M_{s}}\frac{1}{t_{\mathrm{NiFe}}}(1-e^{\frac{-2t_{{\mathrm{WTe}}_{2}}}{\lambda_{\mathrm{SD}}}})$$

**Figure 4:**
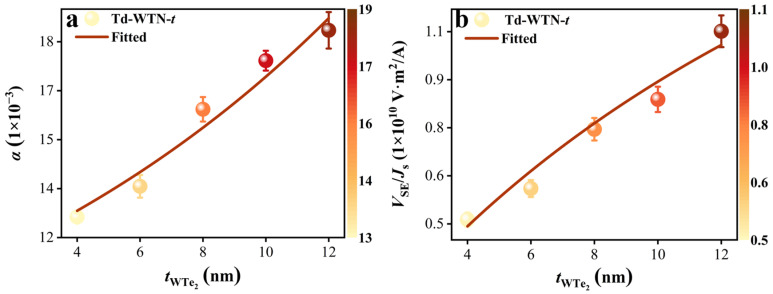
(**a**) Dependence of the *α* in Td-WTN-*t* samples on the thickness of Td-WTe_2_. (**b**) The correlation between *V*_SE_/$J_{S}$ and $t_{{\mathrm{WTe}}_{2}}$ in Td-WTN-*t* samples.

Here, the spin backflow at the Td-WTe_2_/NiFe interface is reflected by the exponential term, and $g_{r}^{↑↓}$ denotes the spin-mixing conductance of Td-WTN-*t*, incorporating the spin backflow. Figure 4a shows the exponential relationship between the $t_{{\mathrm{WTe}}_{2}}$ and *α* in the Td-WTN-*t* samples, indicating that the spin backflow recovers part of the angular momentum loss in the NiFe. By fitting the experimental data in Figure 4a using Equation (8), the spin diffusion length *λ*_SD_ = 14.05 ± 6.2 nm was determined, which provides direct evidence for the prominent role of spin backflow effects at the interface.

To rigorously validate the extracted *λ*_SD_ in polycrystalline Td-WTe_2_, we first quantify the spin current density ($J_{S}$) in the Td-WTN-*t* samples using the following expression [54]:

(9) $$J_{S}=\frac{g_{\mathrm{eff}}^{↑↓}2e{\left(\gamma \mu_{0}h_{\mathrm{rf}}\right)}^{2}[\mu_{0}M_{s}\gamma +\sqrt{{\left(\mu_{0}M_{s}\gamma \right)}^{2}+16{\left(\pi f\right)}^{2}}]}{8\pi \alpha^{2}[{\left(\mu_{0}M_{s}\gamma \right)}^{2}+16{\left(\pi f\right)}^{2}]}$$

Here, *f* is set at 4 GHz and *h*_rf_ denotes the *I*_rf_ field (0.017 mT) [40]. Table 1 summarizes the $J_{S}$ for all Td-WTN-*t* samples, demonstrating a gradual increase in $J_{S}$ with increasing Td-WTe_2_ thickness. Furthermore, by fitting the functional relationship between *V*_SE_/$J_{S}$ and $t_{{\mathrm{WTe}}_{2}}$ using Equation (10), *λ*_SD_ is extracted, as shown below [55]:

(10) $$V_{\mathrm{SE}}=\frac{w\theta_{\mathrm{SHE}}\lambda_{\mathrm{SD}}\tanh(\frac{t_{{\mathrm{WTe}}_{2}}}{2\lambda_{\mathrm{SD}}})}{t_{{\mathrm{WTe}}_{2}}\sigma_{{\mathrm{WTe}}_{2}}+t_{\mathrm{NiFe}}\sigma_{\mathrm{NiFe}}}J_{S}$$

where *w* is the sample width 7 mm, *θ*_SH_ is the spin Hall angle, and $\sigma_{{\mathrm{WTe}}_{2}}$ and $\sigma_{\mathrm{NiFe}}$ denote the electrical conductivities of the Td-WTe_2_ and NiFe (see Table S1). The relationship between *V*_SE_/$J_{S}$ and the thickness of Td-WTe_2_ for all Td-WTN-*t* samples was fitted using Equation (10), resulting in a spin diffusion length of *λ*_SD_ = 14.19 ± 1.12 nm, as shown in Figure 4a. This is consistent with our previous findings obtained by fitting the relationship between the *α* of Td-WTN-*t* and the thickness of Td-WTe_2_. Therefore, the *λ*_SD_ of polycrystalline Td-WTe_2_ prepared by CVD is approximately 14 nm, further confirming its accuracy in comparison to the reported *λ*_SD_ values of other TMDs along the thickness direction [40,49]. Table 1 shows that Td-WTe_2_ films with thicknesses of 4–12 nm exhibit *θ*_SH_ ranging from 0.0785 to 0.096, comparable to heavy metals like Ta and Pt [56,57]. This highlights the effective spin-to-charge conversion capability of polycrystalline few-layer Td-WTe_2_, where *θ*_SH_ exhibits a thickness-dependent enhancement, suggesting a dominant contribution from the bulk ISHE. Figure S3b shows the relationship between the *V*_SE_ and input power (*P*) for Td-WTN-4 in the range of 18–25 dBm at 4 GHz. The results reveal that *V*_SE_ decreases linearly with reduced power, as further demonstrated by the linear fitting in Figure S3c.

**Figure 5:**
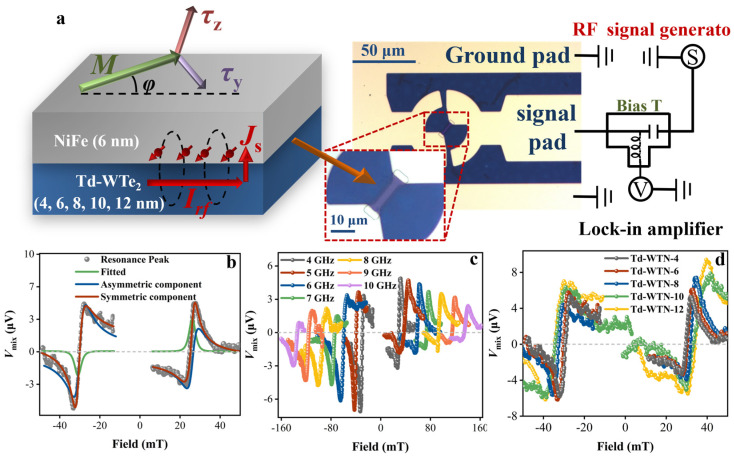
(**a**) Schematic of the ST-FMR test equipment unit and the Td-WTN-4 device. (**b**) ST-FMR results of Td-WTN-4 at 4 GHz. *V*_S_ and *V*_A_ are symmetric and antisymmetric fitted voltages for *V*_mix_. (**c**) ST-FMR spectrum of Td-WTN-4 in the 4–10 GHz range. (**d**) ST-FMR spectra of Td-WTN-*t* (4, 6, 8, 10, and 12 nm) at 4 GHz.

We employed the ST-FMR technique to systematically characterize SOT in a polycrystalline few-layer Td-WTe_2_ spin source layer, and the specific test equipment is shown in Figure 5a. At room temperature, when *I*_rf_ is injected into the Td-WTe_2_ layer, a spin current is generated via the SHE or REE and injected into the adjacent NiFe layer. This induces an SOT, driving the precessional dynamics of the NiFe magnetic moments. When the *f* of *I*_rf_ matches the resonance *f* of NiFe, the lock-in amplifier detects the resistance oscillation signal *V*_mix_ induced by ferromagnetic resonance, while the optimization of resonance conditions is achieved by scanning the magnetization direction at an angle of *φ* = 45° relative to the direction of *I*_rf_. The SOT generated by the Td-WTe_2_ layer comprises an in-plane damping-like torque ($\tau_{\mathrm{DL}}$ = **m × z × m**) and an out-of-plane field-like torque ($\tau_{\mathrm{FL}}$ = **z × m**), where **m** is the magnetization vector of the NiFe layer, and **z** denotes the induced spin polarization [58,59,60]. Additionally, the inset in Figure 5a presents the MO image of the fabricated lithographic device. Figure 5b presents the characteristic *V*_mix_ signals of Td-WTN-4 under both positive and negative external magnetic fields at a frequency of 4 GHz (25 dBm). Utilizing Equation (11), the symmetric Lorentzian voltage *V*_S_ (green line) and asymmetric Lorentzian voltage *V*_A_ (blue line) components were extracted from the *V*_mix_ signals through fitting, as demonstrated in the following analysis [25].

(11) $$V_{\mathrm{mix}}=\frac{V_{S}\Delta H^{2}}{\Delta H^{2}+{\left(H_{\mathrm{ext}}-H_{0}\right)}^{2}}+\frac{V_{A}(H_{\mathrm{ext}}-H_{0})\Delta H}{\Delta H^{2}+{\left(H_{\mathrm{ext}}-H_{0}\right)}^{2}}$$

In conventional heavy metals, *V*_S_ and *V*_A_ represent damping-like and field-like SOT components, while the reduced crystal symmetry of Td-WTe_2_ can induce additional torque contributions from symmetry-breaking spin-orbit interactions. Furthermore, the characteristic *V*_mix_ spectra of Td-WTN-4, obtained under magnetic field sweeps at 4–10 GHz (Figure 5c), demonstrate that the amplitude of *V*_mix_ decreases with increasing *f*. Figure 5d presents the *V*_mix_ signal of Td-WTN-*t*, revealing its significant dependence on the thickness of Td-WTe_2_. This phenomenon can be attributed to the key role of the bulk SHE in the charge–spin transition process. Figure S4a summarizes the *H*_0_ positions of Td-WTN-*t* at different *f*, with the *M*_eff_ obtained by fitting with Equation (5), yielding a value close to *M*_s_ (see Table 2). As $t_{{\mathrm{WTe}}_{2}}$ increases, the bulk SHE in Td-WTN-*t* becomes increasingly dominant, which enhances magnetic anisotropy and consequently leads to a decrease in *M*_eff_. To extract the *α* parameter (Td-WTN-*t*) in ST-FMR tests, the following formula can be used for fitting [61].

(12) $$\mu_{0}\Delta H=\mu_{0}\Delta H_{0}+\frac{2\pi }{\gamma }\alpha f$$

We applied Equation (12) to fit the Δ*H*-*f* dependence of the Td-WTN-*t* samples shown in Figure S4b, extracted the corresponding *α* coefficients (see Table 2), and observed a marked increase in *α* with increasing Td-WTe_2_ thickness. This increased *α* suggests greater dissipation of spin angular momentum in thicker Td-WTe_2_ layers. Furthermore, the small linewidth of Δ*H*_0_ observed in all samples provides further evidence of the uniform deposition of polycrystalline few-layer Td-WTe_2_ films on the SiO_2_ (300 nm)/Si substrate.

To investigate the unconventional SOT induced by spin currents in the Td-WTe_2_ layer, we conducted *φ*-dependent measurements (*φ* = 0–360°) of the ST-FMR voltage signals for the Td-WTN-*t* samples under an in-plane magnetic field. The *V*_S_ and antisymmetric *V*_A_ components were analyzed by fitting them according to Equations (13) and (14), enabling the extraction of relevant parameters [62].

(13) $$V_{S}=(V_{S,x}\sin(\varphi +\varphi_{0})+V_{S,y}\cos(\varphi +\varphi_{0})+V_{S,z})\sin(2(\varphi +\varphi_{0}))$$

(14) $$V_{A}=(V_{A,x}\sin(\varphi +\varphi_{0})+V_{A,y}\cos(\varphi +\varphi_{0})+V_{A,z})\sin(2(\varphi +\varphi_{0}))$$

**Figure 6:**
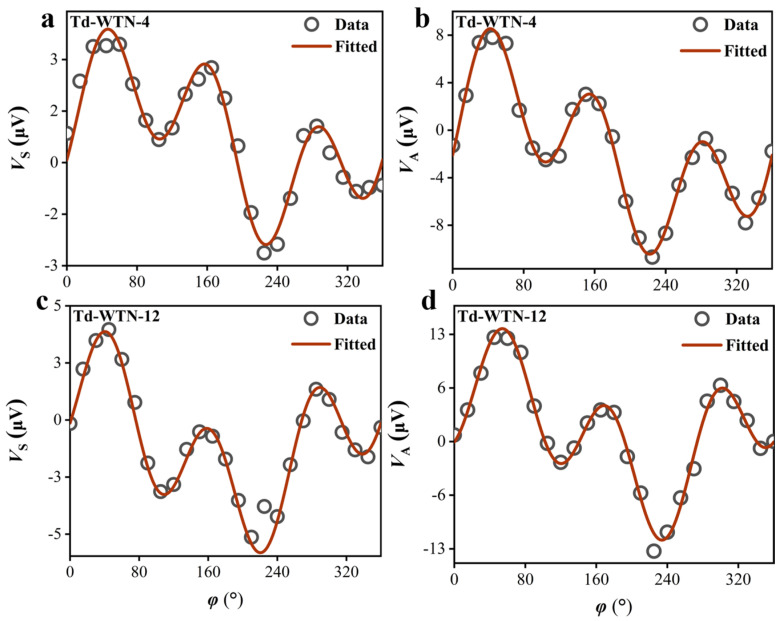
(**a**,**b**) The angular dependence of the ST-FMR signals with plane magnetic field for *V*_S_ and *V*_A_ components of Td-WTN-4 at 4 GHz. (**c**,**d**) The angular dependence of the in-plane magnetic field on the *V*_S_ and *V*_A_ components of Td-WTN-12 at 4 GHz.

Here, *φ*_0_ denotes the angular calibration offset; *V*_S,x_ and *V*_S,y_ originate from the in-plane $\tau_{\mathrm{DL}}$, whereas *V*_S,z_ corresponds to the in-plane $\tau_{\mathrm{FL}}$; *V*_A,x_ represents the out-of-plane $\tau_{\mathrm{FL}}$; *V*_A,y_ originates from the out-of-plane torque induced by the Oersted field generated by the *I*_rf_; and *V*_A,z_ corresponds to the out-of-plane $\tau_{\mathrm{DL}}$. Figure 6a,b and Figure S5a–f present the angular-dependent behavior of the *V*_A_ and *V*_S_ components for Td-WTN-*t* samples with varying thicknesses at a frequency of *f* = 4 GHz, accompanied by the corresponding fitting curves derived from Equations (13) and (14). Also, the amplitudes of the relevant *V*_S_ and *V*_A_ components extracted from all samples were used to systematically study the efficiency of conventional and unconventional SOT. Since the fitting parameters *V*_A,x_ and *V*_S,x_ exhibit negligible magnitudes, the contribution of the spin currents generated along the x-direction can be considered insignificant.

**Figure 7:**
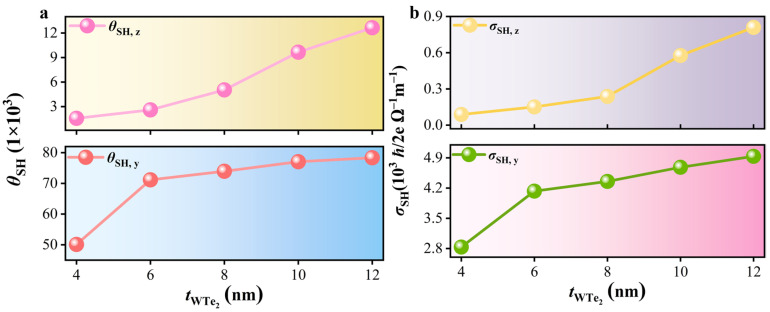
(**a**) The *θ*_SH,y_ and *θ*_SH,z_ of the Td-WTN-*t* heterostructure vary as functions of $t_{{\mathrm{WTe}}_{2}}$. (**b**) The *σ*_SH,y_ and *σ*_SH,z_ of the Td-WTN-*t* heterostructure as a function of $t_{{\mathrm{WTe}}_{2}}$.

Based on the out-of-plane $\tau_{\mathrm{FL}}$ effects primarily originating from the Oersted field contribution induced by the *I*_rf_ flowing through the Td-WTe_2_ layer, this study employs the correlation between the *V*_A,y_ and the current density vector in the Td-WTN-*t* heterostructure. Through standardized normalization, we quantitatively characterize *θ*_SH_. Therefore, based on the extracted amplitudes of *V*_S,y_, *V*_A,y_ and *V*_A,z_, the effective spin Hall angles for the in-plane *θ*_SH,y_ and out-of-plane *θ*_SH,z_ components were quantified using Equations (15) and (16), expressed as [59]

(15) $$\theta_{\mathrm{SH},y}={\left(1+M_{\mathrm{eff}}/H_{0}\right)}^{1/2}\frac{e\mu_{0}M_{s}t_{{\mathrm{WTe}}_{2}}t_{\mathrm{NiFe}}}{\hbar }\frac{V_{S,y}}{V_{A,y}}$$

(16) $$\theta_{\mathrm{SH},z}=\frac{e\mu_{0}M_{s}t_{{\mathrm{WTe}}_{2}}t_{\mathrm{NiFe}}}{\hbar }\frac{V_{A,z}}{V_{A,y}}$$

where *M*_eff_ represents the effective magnetization of the Td-WTN-*t* samples (see Table 2), while *M*_s_ = 605 kA/m denotes the saturation magnetization of the NiFe layer (see Table 1). Table 2 presents the in-plane *θ*_SH,y_ for Td-WTN-4, Td-WTN-6, Td-WTN-8, Td-WTN-10, and Td-WTN-12 as 0.050, 0.071, 0.074, 0.077, and 0.078, respectively. Notably, the unconventional out-of-plane *θ*_SH,z_ for Td-WTN-*t* (4, 6, 8, 10, and 12 nm) exhibits an increasing trend with Td-WTe_2_ thickness, with corresponding values of 0.002, 0.003, 0.005, 0.010, and 0.013. Figure 7a illustrates the dependence of *θ*_SH,y_ and *θ*_SH,z_ on $t_{{\mathrm{WTe}}_{2}}$, revealing that both *θ*_SH,y_ and *θ*_SH,z_ increase with increasing Td-WTe_2_ thickness. This enhancement can be attributed to the dominant contribution of the bulk SHE, where the amplified bulk SHE facilitates greater spin current accumulation at the interface, thereby augmenting both *θ*_SH,y_ and *θ*_SH,z_. Notably, when *I*_rf_ is applied along the low-symmetry crystallographic direction of Td-WTe_2_, it typically induces an unconventional out-of-plane $\tau_{\mathrm{DL}}$ [23]. However, since the few-layer Td-WTe_2_ fabricated by CVD in our study is polycrystalline, the aforementioned examples cannot be directly invoked to explain the origin of the out-of-plane *θ*_SH,z_. Nevertheless, recent studies by Guimaraes et al. and Bangar et al. on polycrystalline materials suggest that the observed out-of-plane $\tau_{\mathrm{DL}}$ may arise from stress-induced symmetry breaking during the sample fabrication process [20,63].

To determine the spin Hall conductivities (*σ*_SH_) at the interface between polycrystalline few-layer Td-WTe_2_ and NiFe along the y and z components, the *σ*_SH_ can be obtained by normalizing the measured *θ*_SH_ with the *ρ* of the Td-WTe_2_ thin film, as expressed by [64]

(17) $$\sigma_{\mathrm{SH},y(z)}=\theta_{\mathrm{SH},y(z)}/\rho$$

Here, *σ*_SH,y_ and *σ*_SH,z_ represent the *σ*_SH_ along the y- and z-directions, respectively. The *σ*_SH,y_ and *σ*_SH,z_ for Td-WTN-*t* (*t* = 4, 6, 8, 10, and 12 nm) are summarized in Table 2. Notably, *σ*_SH,y_ = 4.93 × 10^3^ (ℏ/2e) Ω^−1^m^−1^ and *σ*_SH,z_ = 0.81 × 10^3^ (ℏ/2e) Ω^−1^m^−1^ are achieved at $t_{{\mathrm{WTe}}_{2}}$ = 12 nm. Due to the polycrystalline nature of the fabricated Td-WTe_2_, both *σ*_SH,y_ and *σ*_SH,z_ are smaller than those of single-crystal Td-WTe_2_ but remain within the same order of magnitude [65]. And these values are comparable to the *σ*_SH_ reported by Shi et al. for epitaxial Td-WTe_2_ thin films grown via CVD [66], demonstrating that our polycrystalline few-layer Td-WTe_2_ also exhibits a significant out-of-plane SOT efficiency. Moreover, the *σ*_SH,y_ and *σ*_SH,z_ of the Td-WTN-*t* samples increase with $t_{{\mathrm{WTe}}_{2}}$, as shown in Figure 7b. This trend further supports the fact that in thicker Td-WTN-*t* devices (when the Td-WTe_2_ thickness is less than *λ*_SD_), the bulk SHE plays a dominant role, enabling a more efficient spin current injection into the NiFe layer, which in turn enhances both *σ*_SH,y_ and *σ*_SH,z_. Furthermore, these findings suggest the potential for tuning the unconventional SOT in polycrystalline Td-WTe_2_ thin films by precisely controlling their thickness.

## 4. Conclusions

In summary, we successfully obtained centimeter-scale polycrystalline Td-WTe_2_ films with controlled thicknesses using CVD. The anisotropic scattering of charge carriers in Td-WTe_2_ with varying thicknesses leads to consistently higher MR under out-of-plane magnetic fields compared to in-plane configurations. Thickness-resolved spin pumping in Td-WTN-*t* identifies bulk ISHE as the primary spin–charge conversion mechanism. By modeling *α* and *V*_SE_/$J_{S}$ vs. $t_{{\mathrm{WTe}}_{2}}$, we extract *λ*_SD_ ≈ 14 nm, a critical parameter for vertical spintronic devices. Through angle-resolved ST-FMR, we resolve the $t_{{\mathrm{WTe}}_{2}}$ dependence of both in-plane and out-of-plan $\tau_{\mathrm{DL}}$ spin Hall conductivities in Td-WTN-*t* heterostructures, establishing polycrystalline few-layer Td-WTe_2_ as a platform for tunable unconventional SOT via scalable thickness modulation. Thicker Td-WTe_2_ boosts bulk SHE-driven spin current injection into NiFe, enhancing both in-plane and out-of-plane $\tau_{\mathrm{DL}}$. Consequently, achieving controllable out-of-plane spin polarization and field-free magnetization switching in wafer-scale CVD-grown polycrystalline few-layer Td-WTe_2_ films has emerged as a critical research direction for SOT device applications.

## Figures and Tables

**Table 1:** The *M*_s_ of the Td-WTN-*t* heterostructure and NiFe(6), along with the various parameters extracted from the spin pumping measurements of both the Td-WTN-*t* heterostructure and NiFe(6), are presented.

Sample	*M*_s_ (kA/m)	*M*_eff_ (kA/m)	*α*	Δ*α*/10^−3^	$g_{\mathrm{eff}}^{↑↓}$/(10^19^ m^−2^)	*J*_S_ (A/m^2^)	*θ*_SH_
NiFe(6)	605	522	0.01089	—	—	—	—
Td-WTN-4	552	549	0.01276	1.87	0.400	3918.63	0.0785
Td-WTN-6	544	547	0.01357	2.68	0.564	4965.47	0.0756
Td-WTN-8	537	488	0.01593	5.04	1.048	6776.04	0.0744
Td-WTN-10	533	473	0.01742	6.53	1.347	7341.57	0.0779
Td-WTN-12	522	454	0.01835	7.46	1.508	7558.32	0.0960

**Table 2:** Various parameters of the Td-WTN-*t* samples extracted from the ST-FMR measurements.

Sample	*M*_eff_ (kA/m)	*α*	*σ*_SH, y_ (10^3^ *ħ*/2e Ω^−1^m^–1^)	*σ*_SH, z_ (10^3^ *ħ*/2e Ω^−1^m^–1^)	*θ*_SH, y_	*θ*_SH, z_
Td-WTN-4	517	0.01266	2.83	0.09	0.050	0.002
Td-WTN-6	510	0.01286	4.13	0.15	0.071	0.003
Td-WTN-8	498	0.01569	4.35	0.24	0.074	0.005
Td-WTN-10	470	0.02249	4.68	0.58	0.077	0.010
Td-WTN-12	441	0.02426	4.93	0.81	0.078	0.013

## Data Availability

Data will be made available on request.

## Supplementary Materials

The following supporting information can be downloaded at https://www.mdpi.com/article/10.3390/nano15100762/s1, Figure S1: (a) Schematic diagram of the growth mechanism of Td-WTe_2_ utilizing WO_x_ (*x* < 3) as the precursor. (b,c) The MO and AFM images of Td-WTe_2_ (4 nm). (d,e) The XPS spectrum of Td-WTe_2_ (4 nm). Figure S2: (a) The VSM diagrams for NiFe(6), Td-WTe_2_(4)/NiFe(6), Td-WTe_2_(4)/NiFe(6)/MgO(2) and Td-WTe_2_(4)/NiFe(6)/MgO(2)/Ti(2), respectively. (b) The XRR spectra (intensity vs. 2*θ*) of Td-WTe_2_(6)/NiFe(6) films. Figure S3: (a) The *V*_s_ of the Td-WTN-*t* samples at 4 GHz. (b) The *V*_SE_ plot of Td-WTN-4 at 4 GHz, ranging from 18 to 25 dBm. (c) The linear relationship between *V*_SE_ and *P* for Td-WTN-4. Figure S4: (a) *H*_0_ dependence of *f* for Td-WTN-*t*. (b) *f* dependence of Δ*H* for Td-WTN-*t*. Figure S5: (a–f) The angular dependence of the ST-FMR signals with plane magnetic field for *V*_S_ and *V*_A_ components of Td-WTN-*t* (6, 8 and 10 nm) at 4 GHz. Table S1: The resistivity (*ρ*_0_) and conductivity (σ) of Td-WTe_2_(*t*) and NiFe(6) samples.
