# Supplementary material for: Observation of Thickness-Modulated Out-of-Plane Spin–Orbit Torque in Polycrystalline Few-Layer Td-WTe2 Film
Source: Nanomaterials (Basel). 2025 May 19;15(10):762. doi: 10.3390/nano15100762 (PMC12113999; doi:10.3390/nano15100762)
Supplement: Supplementary file 1 [file nanomaterials-15-00762-s001.zip › nanomaterials-3609992-supplementary.pdf]

Supporting Documents

Observation of thickness-modulated out-of-plane spin-orbit torque in polycrystalline few-layer Td-WTe_2 film

Mingkun Zheng [a b], Wancheng Zhang [a b], You Lv [c], Yong Liu [d], Rui Xiong [d], Zhenhua Zhang [a b *], and Zhihong Lu [a b *]

[a] State Key Laboratory of Advanced Refractories, Wuhan University of Science and Technology, Wuhan 430081, People's Republic of China

[b] School of Materials, Wuhan University of Science and Technology, Wuhan 430081, People's Republic of China

[c] State Key Laboratory of Rare Earth Resource Utilization, Changchun Institute of Applied Chemistry, Chinese Academy of Sciences, Changchun 130022, People's Republic of China

[d] Key Laboratory of Artificial Micro- and Nano-structures of Ministry of Education, School of Physics and Technology, Wuhan University, Wuhan 430072, People's Republic of China

[*] Corresponding author: Zhenhua Zhang, zzhua@wust.edu.cn

[*] Corresponding author: Zhihong Lu, zludavid@live.com

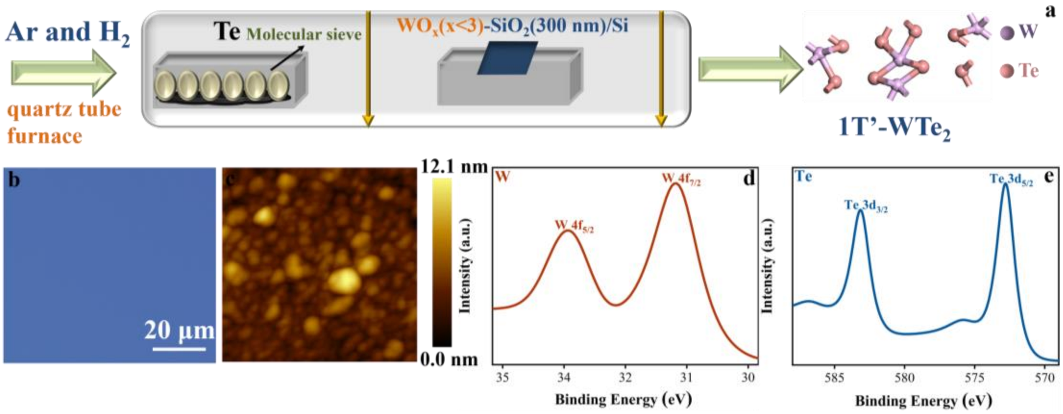

Fig. S1. (a) Schematic diagram of the growth mechanism of Td-WTe_2 utilizing WO_x (x<3) as the precursor. (b) The AFM images of Td-WTe_2 (4 nm). (b, c) The XPS spectrum of Td-WTe_2 (4 nm).

Table S1. The resistivity (ρ_0) and conductivity (σ) of Td-WTe_2(t) and NiFe(6) samples.

**Samples**	**ρ_0 ($10^{-5}\,\Omega \cdot m$)**	**σ ($10^5\,\Omega^{-1}m^{-1}$)**
NiFe(6)	0.05	21.83
WTe_2(4)	1.77	0.57
WTe_2(6)	1.72	0.58
WTe_2(8)	1.70	0.59
WTe_2(10)	1.67	0.60
WTe_2(12)	1.56	0.64

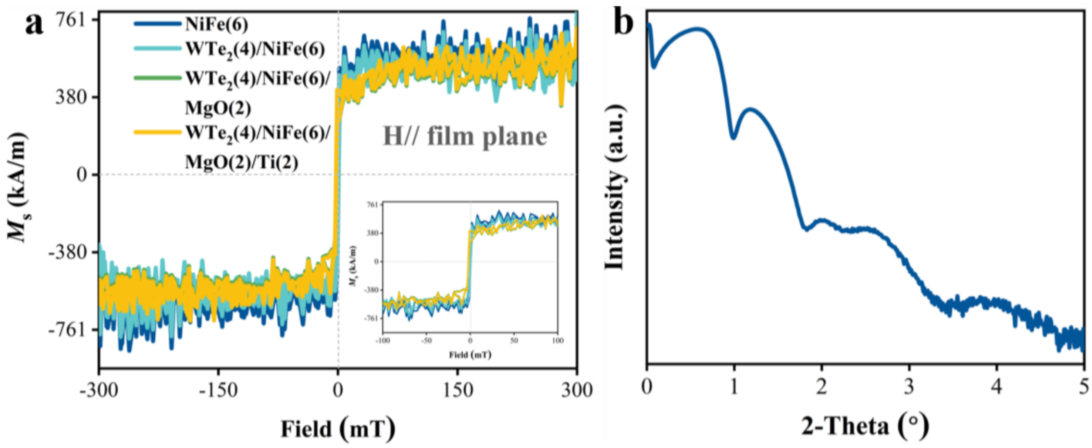

Fig. S2 (a) The VSM diagrams for NiFe(6), Td-WTe_2(4)/NiFe(6), Td-WTe_2(4)/NiFe(6)/MgO(2) and Td-WTe_2(4)/NiFe(6)/MgO(2)/Ti(2), respectively. (b) The XRR spectra (intensity vs. 2θ) of Td-WTe_2(6)/NiFe(6) films.

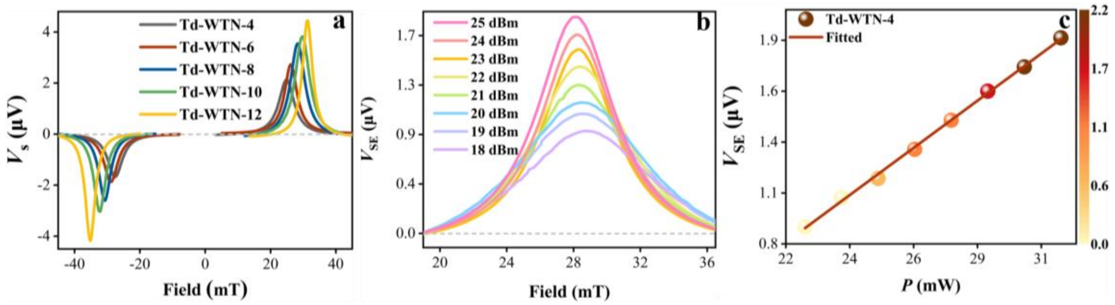

Fig. S3. (a) The V_s of the Td-WTN-t samples at 4 GHz. (b) The V_{SE} plot of Td-WTN-4 at 4 GHz, ranging from 18 to 25 dBm. (c) The linear relationship between the V_{SE} and P for Td-WTN-4.

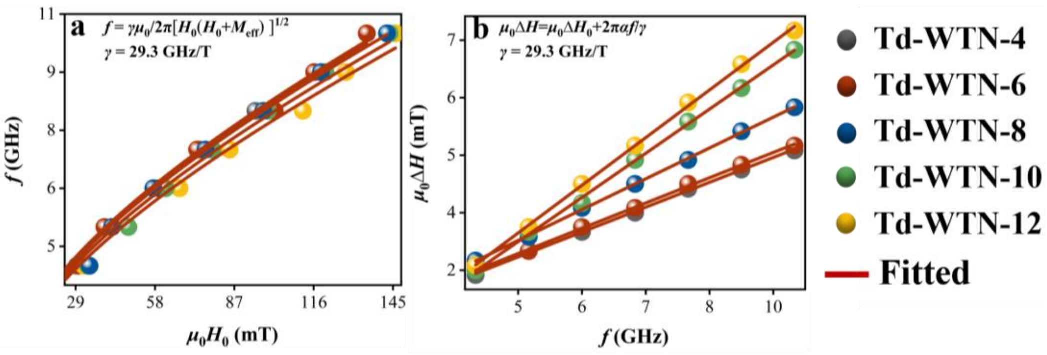

Fig. S4. (a) H_0 dependence of f for Td-WTN-t. (b) f dependence of ΔH for Td-WTN-t.

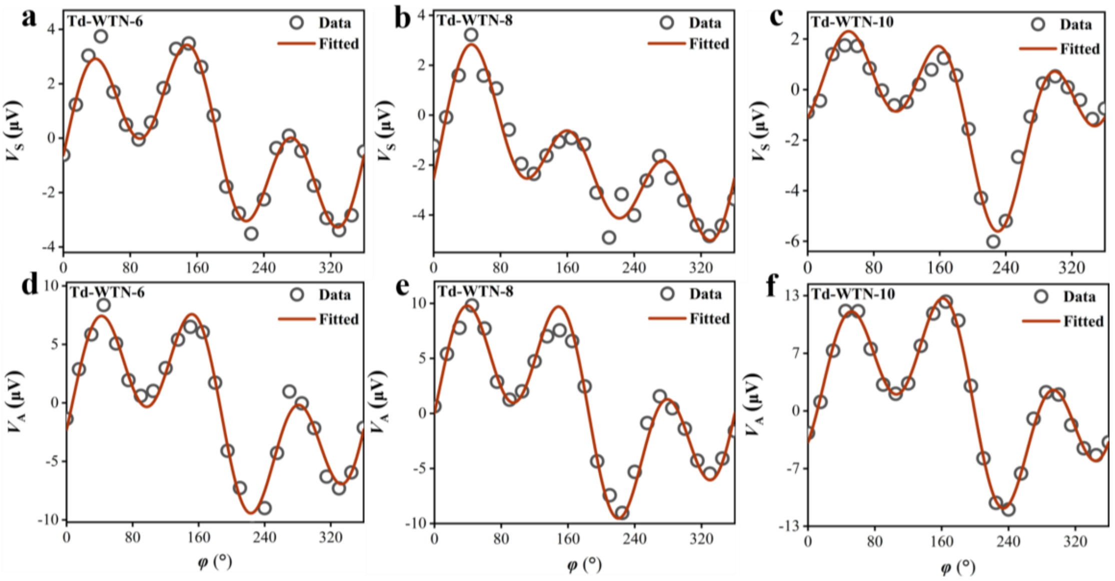

Fig. S5. (a-f) The angular dependence of the ST-FMR signals with plane magnetic field for V_S and V_A components of Td-WTN-t (6, 8 and 10 nm) at 4 GHz.
